# Transcription Factor CHF1/Hey2 Regulates Specific Pathways in Serum Stimulated Primary Cardiac Myocytes: Implications for Cardiac Hypertrophy

**DOI:** 10.2174/138920210791233117

**Published:** 2010-06

**Authors:** Man Yu, Fan Xiang, Richard P Beyer, Federico M Farin, Theo K Bammler, Michael T Chin

**Affiliations:** 1 Division of Cardiology, Department of Medicine, University of Washington, Seattle, WA, USA; 2 Department of Environmental and Occupational Health Sciences, University of Washington, Seattle, WA, USA

**Keywords:** Cardiac hypertrophy, microarray, transcription factor.

## Abstract

We have previously found that overexpression of CHF1/Hey2 in the myocardium prevents the development of phenylephrine-induced hypertrophy. To identify transcriptional pathways regulated by CHF1/Hey2, we cultured primary neonatal mouse cardiac myocytes from wild type and transgenic mice overexpressing CHF1/Hey2 and treated them with serum, a potent hypertrophic stimulus. We verified that overexpression of CHF1/Hey2 suppressed cardiac myocyte hypertrophy induced by serum and then determined transcriptional profiles by microarray hybridization. We identified and verified important downstream target genes by single gene analysis and qRT-PCR and then identified important biological processes by Gene Set Analysis using Biological Process Gene Sets from the Gene Ontology Consortium. We found that CHF1/Hey2 suppresses pathways involved in water transport, adenylate cyclase activity, embryonic eye morphogenesis, gut development and fluid transport after serum stimulation. Genes involved in protein dephosphorylation, demonstrate increased expression in myocytes overexpressing CHF1/Hey2, independent of serum treatment. Genes overexpressed prior to serum treatment are involved in regulation of transcription factor activity, nuclear protein export and steroid hormone receptor signaling. Genes overexpressed after serum treatment are involved in autophagy, apoptosis and mitochondrial biogenesis.

## INTRODUCTION

Cardiac hypertrophy is an important biological process in which individual cardiac myocytes enlarge in response to exogenous stimuli. Enlargement of myocytes is associated with an increased number of sarcomeres, myofibrils and increasing myocyte width, resulting in an increased ventricular wall thickness. Although hypertrophy is initially compensatory, prolonged exposure to hypertrophic stimuli can ultimately lead to contractile dysfunction and apoptotic cell death, resulting in cardiac failure and fibrosis. Heart failure is a leading cause of morbidity and mortality in Western Society, with the incidence increasing every year. Understanding the molecular basis for the development of hypertrophy, its progression to heart failure and the biological pathways involved in this syndrome will have important preventative, diagnostic and therapeutic implications. 

We have previously reported that the Hairy-related bHLH transcriptional repressor, CHF1 (also called Hey2, Hesr-2, Hrt2, HERP1 and gridlock), functions as a suppressor of cardiac hypertrophy induced by phenylephrine [1]. Suppression of cardiac hypertrophy is mediated, at least in part, by the binding of CHF1/Hey2 to the transcription factor. GATA4, thereby inhibiting the ability of GATA4 to activate atrial natriuretic factor (ANF) gene expression along with other genes associated with cardiac hypertrophy [1-3]. CHF1/Hey2 is normally expressed in the developing heart and vasculature and plays a critical role in heart development, possibly as an effector of Notch signaling, as previously described [4-12]. Additional studies have underscored the importance of CHF1/Hey2 in the development of the myocardium, as loss of CHF1/Hey2 leads to a thin walled myocardium [13], cardiomyopathy [10] and ectopic expression of atrial genes in the ventricular myocardium [14, 15].

In order to identify pathways relevant to cardiac hypertrophy in the cardiac myocyte transcriptome that are regulated by CHF1/Hey2, we examined the expression of genes in isolated wild type and transgenic myocytes treated with serum, a potent hypertrophic stimulus. Here we report that CHF1/Hey2 suppresses the adenylate cyclase pathway, genes involved in water transport and activates cellular phosphatases and genes involved in autophagy. These findings suggest important new pathways in regulation of hypertrophy. 

## MATERIALS AND METHODS

### Transgenic Mice, Neonatal Mouse Myocyte Culture and *In Vitro* Hypertrophy Assays

Transgenic mice overexpressing CHF1/Hey2 in the myocardium under the control of the mlc2v promoter and neonatal mouse cardiac myocyte culture was performed as previously described [1]. The purity of each preparation was verified by immunostaining with an antibody against sarcomeric myosin (MF-20, Developmental Studies Hybridoma Bank) to be greater than 95%. To induce hypertrophy, neonatal myocytes in serum free medium were exposed to media with 20% fetal bovine serum for 24-48 hours. Hypertrophy was assessed at 48 hours by immunostaining cells with a monoclonal antibody against sarcomeric myosin (MF-20, Developmental Studies Hybridoma Bank), digital image capture and cell size planimetry as previously described [1]. To identify important transcriptional pathways involved in hypertrophy, RNA was harvested 24 hours after treatment as described below.

### RNA Isolation and Microarray Analysis

RNA was isolated from cultured myocytes by extraction with a commercially available kit (Qiagen) according to the manufacturer’s instructions. For microarray hybridization, RNA was processed for hybridization to Affymetrix GeneChip® Mouse Gene 1.0 ST Arrays through a core facility in the Functional Genomics Laboratory at the Center for Ecogenetics and Environmental Health at the University of Washington, as previously described [16] and according to the manufacturer’s instructions. The number of independent cultures that were harvested for RNA and processed for each condition is as follows: WT no serum, 5; WT with serum, 7; TG no serum, 6; TG with serum, 7.

Raw microarray data was processed with Affymetrix^®^ Expression Console™ Software using Robust Multichip Analysis (RMA). Normalized data was analyzed first by single gene analysis and then by Gene Set Analysis [17], a modification of Gene Set Enrichment Analysis [18]. Selected genes were validated by qRT-PCR as previously described [19]. A full description of the Gene Set Analysis method is available online at http://www-stat.stanford.edu/~tibs/GSA/. Gene sets for analysis were obtained from the Gene Ontology Project (www.geneontology.org). We focused specifically on gene sets organized according to Biological Process (BP; Gene Ontology). Gene sets were considered to be significantly altered when their statistical p values and false discovery rates as determined by the software were <0.001. All microarray data have been deposited in the Gene Expression Omnibus Database under accession number GSE14288.

## RESULTS

### Cardiac Myocytes that Overexpress CHF1/Hey2 are Resistant to Serum Induced Hypertrophy *In Vitro*

We previously reported that overexpression of CHF1/Hey2 in myocytes suppresses hypertrophy induced by phenylephrine *in vivo* and *in vitro* [1]. To verify that this resistance to hypertrophy is a general phenomenon in response to diverse stimuli, we induced hypertrophy of cultured WT and TG cardiac myocytes with 20% serum, a potent inducer of hypertrophy. As shown in Fig.  (**1**), WT myocytes developed increased cell size within 48 hours, as expected. TG myocytes, in contrast, did not develop increased cell size. These findings are consistent with our earlier published results and provide the rationale for the transcriptional profiling studies outlined below. 

### Transcriptional Profiling of Wild Type and Transgenic Myocytes Before and After Serum Stimulation Reveals Alterations in Specific Gene Sets that are Both Serum-Independent and Serum-Dependent

To identify the potential downstream targets of CHF1/Hey2 that may play a role in the myocyte hypertrophic response, we analyzed transcriptional profiles from wild type and transgenic myocytes before and after serum stimulation. Both single gene analysis (SGA) as well as category/pathway analysis was performed. SGA assesses statistical significance of each gene on the array separately. This approach identified only one gene (CHF1/Hey2, p value < 0.0001, log_2_FC = 1.58, ~ 3 fold increased, data not shown) as differentially expressed in unstimulated CHF1/Hey2 transgenic versus wildtype cells. This finding is consistent with our earlier published results that myocardial overexpression of CHF1/Hey2 does not result in any significant baseline phenotype [1]. 

Analysis of gene expression profiles in serum stimulated cells revealed several individual genes that show significantly changed expression, defined as a p value < 0.0001, as shown in Table (**1**). CHF1/Hey2 is significantly changed, as expected. Other genes include Il1f6, Cecr2, Tmc4, Ndufs5, Ndufv3 and Efcab2. IL1F6 is a proinflammatory cytokine, CECR2 is a transcription factor that interacts with chromatin remodeling proteins, while TMC4 is a transmembrane protein of unknown function. NDUFS5 and NDUFV3 are components of the mitochondrial respiratory chain NADH complex I, while EFCAB2 is a putative calcium binding protein of unknown function. To validate some of these findings, we performed qRT-PCR for CHF1/Hey2, Ndufs5, Nudfv3 and Efcab2. As shown in Fig. (**2**), the mRNA levels are accurately reflected by the microarray data. The roles of these potential target genes in cardiac hypertrophy, to the best of our knowledge, are unknown, however.

To gain further insight into potential biological pathways altered in our transgenic cells relative to the wildtype cells, we carried out category/pathway analysis. More specifically, we performed Gene Set Analysis (GSA) [17]. In contrast to SGA, GSA assesses statistical significance of a gene set/pathway as a whole. This analysis allows for identification of gene sets/pathways with genes that show coordinated changes in expression, even if the changes are modest. SGA and GSA are two complementary approaches. SGA identifies any transcript that meets a chosen filtering criteria such as a specific p-value cutoff independent of a pathway content, whereas GSA identifies gene sets/pathways with genes that share a common functional theme. We examined gene sets organized by Gene Ontology Biological Process (BP). Table **2**. lists gene sets with p<0.001. GSA reveals suppression of gene sets involved in embryonic eye morphogenesis, gut development, water transport, fluid transport and regulation of adenylate cyclase activity in the transgenic cells, after serum stimulation. Components of these gene sets may be direct targets of transcriptional repression by CHF1/Hey2. 

Analysis of gene sets that are increased in transgenic relative to WT cells also revealed a number of interesting pathways. In the absence of serum, gene sets involved in regulation of transcription factor activity, protein export from the nucleus, steroid hormone receptor signaling and regulation of protein phosphatase activity are increased. Similar gene sets involved in protein dephosphorylation and dephosphorylation are increased both in the absence and presence of serum. The individual components of these related gene sets overlap significantly, and their significance scores are largely driven by the same genes. In the presence of serum, multiple gene sets involved in autophagy, apoptosis, mitochondrial biogenesis, cellular component disassembly and viral reproduction demonstrate increased expression. Although CHF1/Hey2 is considered to be a transcriptional repressor, activation of multiple pathways may reflect either positive activation by CHF1/Hey2 or repression of negative regulatory factors for these pathways.

Analysis of individual components from various BP gene sets suppressed by CHF1/Hey2 reveals multiple potential targets that may play a role in hypertrophy. These include genes involved in water transport (GO:0006833) such as aquaporins 2, 3, 5, 6, 7, 8, 9 and 11, suggesting that CHF1/Hey2 globally suppresses water transport and that water transport may be important for cardiac hypertrophy (Fig. (**3**)). In addition, aquaporins 3, 7 and 9 also transport glycerol and are known as aquaglyceroporins. AQ7 null mice develop adipocyte hypertrophy, obesity and diabetes due to impaired glycerol efflux [20-22]. These findings raise the intriguing possibility that suppression of glycerol efflux in cardiac myocytes may prevent the onset of pathological hypertrophy, perhaps through supporting fatty acid metabolism. Genes involved in regulation of adenylate cyclase signaling (GO:0045761, (Fig. (**4**)) include multiple G protein coupled receptors such as ADCYAP1R1, LTB4R2, ADRB2, GABBR1, GALR3, GHRHR, CRHR1, ADRA2A, VIPR2, GRM4 and intracellular regulators of the adenylate cyclase response such as ADCY4, GNAL, ADCY2, ADCY7, ADCY1. Given the known role of adrenergic signaling in the pathogenesis of cardiac hypertrophy and heart failure (reviewed in [23]), suppression of adenylate cyclase activity is consistent with these findings.

Analysis of individual components of other gene sets suppressed in transgenic myocytes after serum stimulation reveals a number of interesting regulatory genes. Genes involved in eye development (GO: 0048048) include the transcription factors VAX2, RARG, FOXL2 and SP1 and the metabolic enzymes ALDH1A1 and ALDH1A3. Genes involved in gut development (GO: 0048565) include many genes known to be important in developmental signaling and transcription such as TCF7, NOTCH1, NKX2-3, HOXD13, GLI2, FOXF2 and SHH (Fig. (**5**)). Genes involved in fluid transport (GO: 0042044) include the aquaporins described above for GO: 0006833 and are largely redundant. A role for any of these molecules in cardiac hypertrophy and heart failure, to the best of our knowledge, has not yet been described.

Analysis of individual components of gene sets that are activated in the CHF1/Hey2 transgenic myocytes in both the absence or presence of serum (GO: 0006470, GO: 0016311) reveals a number of phosphatases, such as PTPN4, DUSP28, MTMR6, PPP3R1, DUSP8, PPP6C, PPM1B, PPP2R4, PPP2R2A and PTPN2 Fig. (**6**)). DUSP8 and PPM1B can turn off components of the cellular stress response [24, 25], while PTPN2 can attenuate EGF signaling [24, 25], both which may be relevant to hypertrophy.

Analysis of individual components of gene sets that are activated in the CHF1/Hey2 transgenic myocytes only in the absence of serum reveals a number of interesting proteins that regulate the function and/or transport of other proteins. Genes involved in regulation of transcription factor activity (GO: 0051090) include UBE2N, SUMO1, ABRA, COMMD7, PRDX3, JMY and MALT1. These molecules can regulate the activity of important transcriptional activators or coactivators, such as p53 (SUMO1), HIF1α (SUMO1), SRF (ABRA), p300 (JMY) and NF-kB (UBE2N, COMMD7, PRDX3, MALT1). Genes involved in protein export from the nucleus (GO: 0006611) include XPO7, CALR, GSK3B, PRKACA, PTPN11 and DUSP16. XPO7 is involved in general export of nuclear proteins, CALR is involved in nuclear export of steroid hormone receptors, while GSK3B, PRKACA, PTPN11 and DUSP16 are also involved in signal transduction as kinases (GSK3B, PRKACA) or phosphatases (PTPN11, DUSP16). Genes involved in steroid hormone receptor signaling (GO: 0030518) include MED1, JMJD1A, PIAS1, NR3C2, JAK2, MED30, PGR, MED14 and PPARGC1A. Genes involved in regulation of phosphoprotein phosphatase activity include PPP2R4, SAPS2, JAK2, SAPS1, SAPS3, RCAN1 and PPP1R2. Increased expression of these various genes only in the absence of serum suggests differences in baseline metabolism of the transgenic myocytes, however, their role in cardiac myocyte hypertrophy is unclear, as the differences in expression do not persist after serum stimulation.

Analysis of individual components of gene sets that are activated in the CHF1/Hey2 transgenic myocytes after serum stimulation reveals a surprising number of genes that play roles in autophagy and apoptosis. Genes involved in autophagy (GO: 0016236, GO: 0000045) include ATG4A, ATG9A, MAPILC3A, ATG4C, ATG16L1, FRAP1, ATG5, and ATG4D (Fig. (**7**)). Genes involved in apoptosis and cellular component disassembly (GO: 0006309, GO: 0006921, GO: 0030262, GO: 0022411) include CECR2, CIDEA, CYCS, DFFA, BNIP3 and AIFM1 (Fig. (**8**)). Genes involved in mitochondrial biogenesis (GO: 0007005) include NDUFS5, DAP3, VDAC1, DNAJC19, MTX2, MIPEP, DCTN6, AIFM2, AIP, FXN, DNAJ3, BNIP3, IMMP2L, AIFM1, TIMM8B, TIMM10, TIMM9 and NDUFS8 (Fig. (**9**)). Genes involved in viral reproduction (GO: 0022415) include HBXIP, TNIP1, HIPK2, INSR, FURIN, XRCC4, XRCC6, CCL5, USF1, WWP1, ITCH, WWP2, NFIA, LIG4 and SMAD3. Activation of autophagy genes is interesting, and is consistent with a protective role for autophagy in the heart [26, 27], as previously suggested. Activation of apoptotic genes is paradoxical, as the transgenic hearts are known to be resistant to hypertrophy and apoptosis [28], and may reflect a homeostatic feedback loop in response to a downstream block to apoptosis.

## DISCUSSION

We have previously found that CHF1/Hey2 overexpression in the myocardium blunts the hypertrophic response induced by phenylephrine both *in vivo* and *in vitro* [1]. To further delineate transcriptional pathways regulated by CHF1/Hey2 specifically in cardiac myocytes that are relevant to hypertrophy, we cultured cardiac myocytes from wild type and transgenic mice and treated them with serum. As expected, transgenic overexpression of CHF1/Hey2 prevented the development of hypertrophy induced by serum. We next compared their transcriptomes before and after serum treatment. As described above, we have found that CHF1/Hey2 regulates a number of gene sets that may play important roles in cardiac hypertrophy. 

The identification of specific biological processes altered by CHF1/Hey2 through Gene Set Analysis reveals both previously identified and novel pathways that likely affect the development of hypertrophy. Numerous microarray studies examining the transcriptional profiles in various models of hypertrophy have been performed, but consensus findings have been limited (reviewed in [23]). No specific molecular signature for hypertrophy has been described, as transcriptional profiles are divergent in different models of hypertrophy [29]. The advantage of Gene Set Analysis and its relative, Gene Set Enrichment Analysis, is that it more readily detects small concordant changes in the expression of genes acting in concert, which may alter the flux through a pathway more readily than a more dramatic change in a single gene [18]. A caveat to this method is that the individual genes within a gene set may not have significantly different gene expression by single gene analysis, and therefore the relevance of gene components within each set cannot easily be validated. Nevertheless, the method is useful for generating hypotheses for further experimentation. Using this method, we have identified potential roles for genes involved in water transport, regulation of adenylate cyclase activity, protein dephosphorylation, embryonic development, apoptosis and mitochondrial biogenesis, although the exact mechanisms by which these gene products may affect hypertrophy will require further study. 

**Fig. (8):**
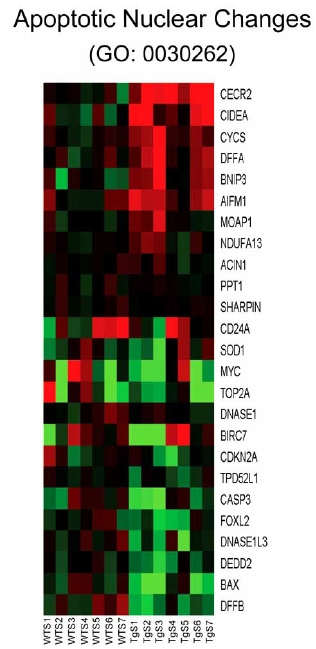
Heat map for genes pertaining to apoptotic nuclear changes (GO: 0030262). Columns represent microarray data obtained from independent cultures of cells of the indicated genotype and therefore represent biological replicates. Gene name abbreviations for each component of the gene set are indicated at the end of each row. Green indicates expression is lower, while red indicates expression is higher than average of WT. The genes in this set are predominantly upregulated in the transgenic cells (TgS1/2/3/4/5/6/ 7) in the presence of serum relative to the WT cells treated with serum (WTS1/2/3/4/5/6/7).

A surprising finding of our study is that many genes associated with autophagy and apoptosis demonstrate increased expression in the transgenic cells after serum stimulation. Autophagy has recently been reported to promote heart failure [30], although this finding is controversial and directly contradicted by other studies which suggests a protective role for autophagy in cardiac hypertrophy and progression to heart failure [26, 27]. Our data is more consistent with the latter studies. Increased expression of apoptosis related genes is surprising, as we have found that the transgenic cells are resistant to apoptosis induced by pressure overload or hydrogen peroxide treatment [28]. One possible interpretation for this paradoxical finding is that apoptosis is inhibited downstream of these molecules, resulting in a homeostatic feedback loop that leads to enhanced expression of these proximal genes. Examination of the components of these apoptosis related gene sets reveals that the proapoptotic gene BAX is downregulated, even though the majority of the components are upregulated (Fig. **8**). As BAX is a downstream effector of mitochondrion-dependent apoptosis, such a scenario is plausible. Further experimentation will be required to explore this hypothesis.

One limitation of our study is that we do not have a clear idea of the relationship of CHF1/Hey2 to these other genes within the context of a gene network. Although we have considered performing network analysis on our microarray data, we have refrained, as our sample size may not be sufficient in size to conduct a rigorous network analysis with current algorithms. In addition, current algorithms make more assumptions than are present in our current analysis, which may lead to spurious conclusions. In the future, we hope to address this important question as algorithms improve and as we collect more data.

## CONCLUSIONS

We have previously analyzed transcriptional pathways in smooth muscle cells lacking CHF1/Hey2 after growth factor stimulation to gain insight into CHF1/Hey2-dependent genes that determine neointimal formation after vascular injury [16, 31]. We have also previously reported that CHF1/Hey2 can attenuate the hypertrophic response of cardiac myocytes to phenylephrine *in vivo* and *in vitro* [1]. More recently, we have found that CHF1/Hey2 suppresses the development of pathological hypertrophy and promotes physiological hypertrophy through suppression of apoptosis [28]. Our current study examines transcriptional pathways altered in transgenic myocytes treated with serum, a potent hypertrophic stimulus, and reveals associations between CHF1/Hey2 transcriptional regulation, fluid transport, adenylate cyclase, phosphatase activity and a number of other important pathways. These aggregate findings underscore the importance of CHF1/Hey2 in cardiac hypertrophy, and suggest that future therapies based on CHF1/Hey2 may have important therapeutic implications in preventing hypertrophy and heart failure.

## Figures and Tables

**Fig. (1):**
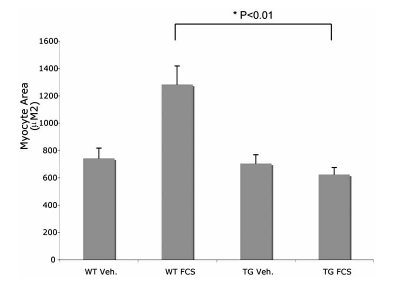
Overexpression of CHF1/Hey2 in cardiac myocytes suppresses serum-induced hypertrophy *in vitro*. Cultured WT and TG myocytes were treated with either vehicle or 20% FCS for 48 hours and then immunostained with MF-20 antibody against sarcomeric myosin to distinguish myocytes from contaminating fibroblasts. Cell area was calculated for 50 cells per experiment for three independent experiments by digital image capture and planimetry. Statistical comparison of average cell size was assessed by a student’s t-test with statistical significance defined as p < 0.05.

**Fig. (2):**
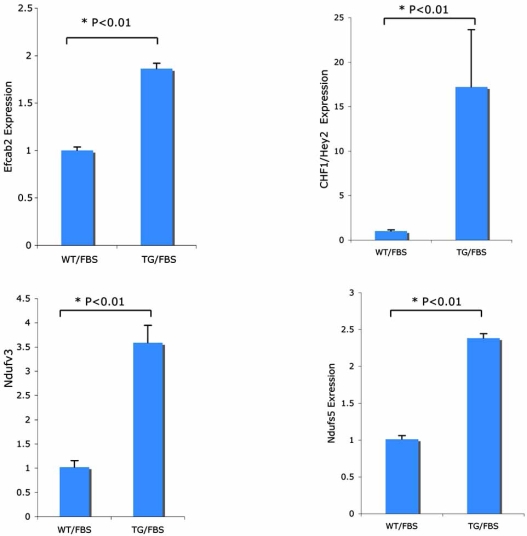
qRT-PCR of transcripts identified by microarray single gene analysis validates their expression profiles. RNA was harvested from multiple independent preparations of WT and TG cells treated with serum, reverse transcribed and amplified by standard qRT-PCR methods. Expression was normalized to GAPDH and compared by the 2^-∆∆C^_T_ method as described [19].

**Fig. (3):**
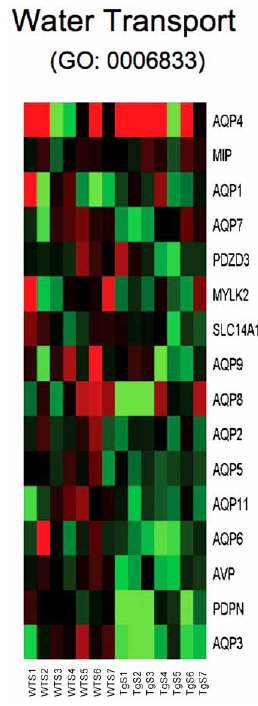
Heat map for genes involved in water transport (GO: 0006833). Columns represent microarray data obtained from independent primary cultures of cells of the indicated genotype and therefore represent biological replicates. Gene name abbreviations for each component of the gene set are indicated at the end of each row. Green indicates expression is lower, while red indicates expression is higher than average of WT. The genes in this set are predominantly downregulated in the transgenic cells (TgS1/2/3/4/5/ 6/7) in the presence of serum relative to the WT cells treated with serum (WTS1/2/3/4/5/6/7).

**Fig. (4):**
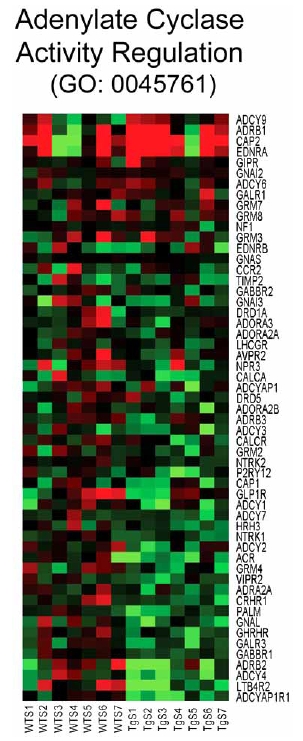
Heat map for genes related to regulation of adenylate cyclase activity (GO: 0045761). Columns represent microarray data obtained from independent primary cultures of cells of the indicated genotype and therefore represent biological replicates. Gene name abbreviations for each component of the gene set are indicated at the end of each row. Green indicates expression is lower, while red indicates expression is higher than average of WT. The genes in this set are predominantly downregulated in the transgenic cells (TgS1/2/3/4/5/6/7) in the presence of serum relative to the WT cells treated with serum (WTS1/2/3/4/5/6/7).

**Fig. (5):**
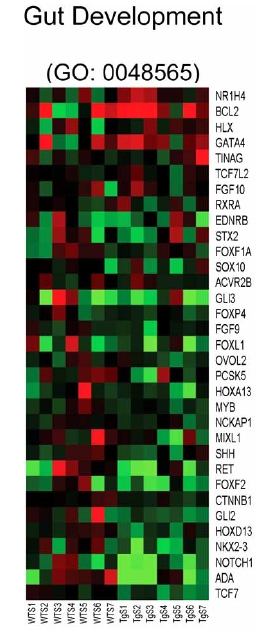
Heat map for genes pertaining to gut development (GO: 0048565). Columns represent microarray data obtained from independent primary cultures of cells of the indicated genotype and therefore represent biological replicates. Gene name abbreviations for each component of the gene set are indicated at the end of each row. Green indicates expression is lower, while red indicates expression is higher than average of WT. The genes in this set are predominantly downregulated in the transgenic cells (TgS1/2/3/4/5/ 6/7) in the presence of serum relative to the WT cells treated with serum (WTS1/2/3/4/5/6/7).

**Fig. (6):**
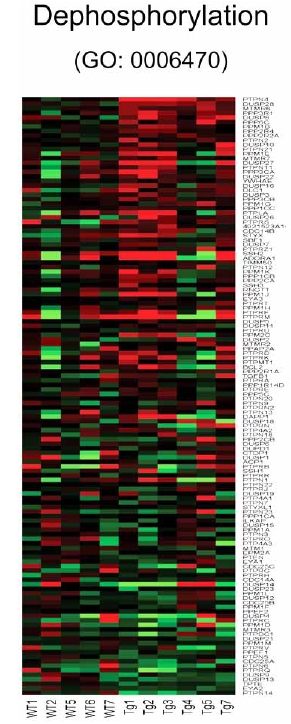
Heat map for genes linked to protein amino acid dephosphorylation (GO: 0006470). Columns represent microarray data obtained from independent primary cultures of cells of the indicated genotype and therefore represent biological replicates. Gene name abbreviations for each component of the gene set are indicated at the end of each row. Green indicates expression is lower, while red indicates expression is higher than average of WT. The genes in this set are predominantly upregulated in the transgenic cells (Tg1/2/3/4/5/7) in the absence of serum relative to the corresponding WT cells (WT 1/2/5/6/7).

**Fig. (7):**
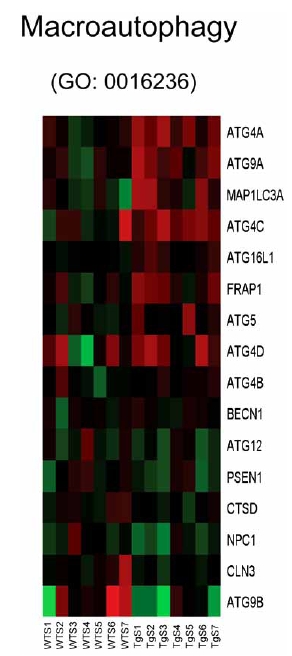
Heat map for genes associated with macroautophagy (GO: 0016236). Columns represent microarray data obtained from independent primary cultures of cells of the indicated genotype and therefore represent biological replicates. Gene name abbreviations for each component of the gene set are indicated at the end of each row. Green indicates expression is lower, while red indicates expression is higher than average of WT. The genes in this set are predominantly upregulated in the transgenic cells (TgS1/2/3/4/5/6/ 7) in the presence of serum relative to the WT cells treated with serum (WTS 1/2/3/4/5/6/7).

**Fig. (9):**
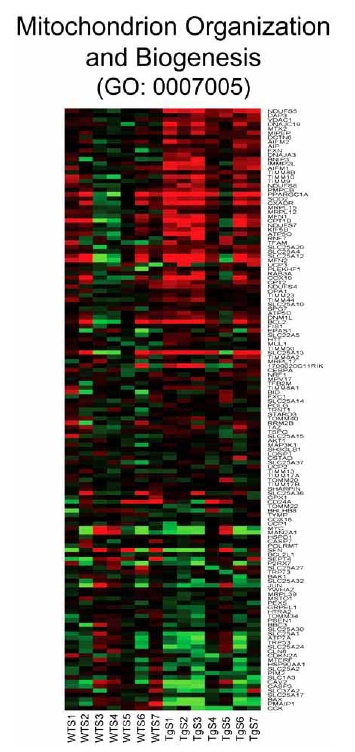
Heat map for genes involved in mitochondrion organization and biogenesis (GO: 0007005). Columns represent microarray data obtained from independent cultures of cells of the indicated genotype and therefore represent biological replicates. Gene name abbreviations for each component of the gene set are indicated at the end of each row. Green indicates expression is lower, while red indicates expression is higher than average of WT. The genes in this set are predominantly upregulated in the transgenic cells (TgS1/2/3/4/5/6/7) in the presence of serum relative to the WT cells treated with serum (WTS1/2/3/4/5/6/7).

**Table 1:** Genes Demonstrating Significant Changes in Expression in CHF1/Hey2 Trangenic Myocytes vs Wild Type myocytes After Serum Stimulation

Gene.Accession	Gene.Symbol	Gene.Description	logFC	P.Value	FDR
NM_013904	Hey2	Hairy/enhancer-of-split related with YRPW motif 2	1.814541571	2.73E-07	0.009724348
NM_019450	Il1f6	Interleukin 1 family, member 6	-0.429964571	1.98E-05	0.117403674
ENSMUST00000112686	Cecr2	Cat eye syndrome chromosome region, candidate 2 homolog (human)	0.437622286	2.26E-05	0.117403674
NM_181820	Tmc4	Transmembrane channel-like gene family 4	-0.306575857	2.31E-05	0.117403674
NM_001030274	Ndufs5	NADH dehydrogenase (ubiquinone) Fe-S protein 5	0.341531429	3.95E-05	0.12082322
NM_030087	Ndufv3	NADH dehydrognase (ubiquinone) flavoprotein 3	0.347003714	6.16E-05	0.12082322
NM_026626	Efcab2	EF-hand calcium binding domain 2	0.473221857	6.46E-05	0.12082322

**Table 2:** Gene Ontology Biological Process (BP) Gene Set Changes in CHF1/Hey2 Transgenic Myocytes vs Wild Type Myocytes before and/or after Serum Stimulation

			Tg *vs.* WT, No Serum			Tg *vs.* WT, Serum		
GO ID	Biological Process	Size	p-value	FDR	Score	p-value	FDR	Score
GO:0006470	protein amino acid dephosphorylation	132	<0.001	<0.001	0.5438348	<0.001	<0.001	0.3885387
GO:0051090	regulation of transcription factor activity	77	<0.001	<0.001	0.4408056	0.465	1.0867179	0.013523
GO:0006611	protein export from nucleus	20	<0.001	<0.001	0.806123	0.147	0.9996068	0.2632729
GO:0030518	steroid hormone receptor signaling pathway	75	<0.001	<0.001	0.3664094	0.159	0.9996068	0.1481744
GO:0043666	regulation of phosphoprotein phosphatase activity	10	<0.001	<0.001	1.2864495	0.081	0.9127665	0.5832501
GO:0016311	dephosphorylation	155	<0.001	<0.001	0.4924526	<0.001	<0.001	0.3715223
GO:0048048	embryonic eye morphogenesis	10	0.126	0.7716088	-0.3379658	<0.001	<0.001	-0.6525282
GO:0048565	gut development	33	0.126	0.7716088	-0.1805751	<0.001	<0.001	-0.6338953
GO:0006833	water transport	16	0.038	0.7716088	-0.8349875	<0.001	<0.001	-1.2825472
GO:0042044	fluid transport	18	0.064	0.7716088	-0.6696553	<0.001	<0.001	-1.1006198
GO:0045761	regulation of adenylate cyclase activity	55	0.019	0.7716088	-0.3603997	<0.001	<0.001	-0.7334642
GO:0016236	macroautophagy	16	0.11	1.0068723	1.0068723	<0.001	<0.001	1.04785
GO:0000045	autophagosome formation	13	0.074	0.9947778	0.5173092	<0.001	<0.001	1.223408
GO:0006309	DNA fragmentation during apoptosis	17	0.337	0.8456392	-0.1261624	<0.001	<0.001	0.9596088
GO:0006921	cell structure disassembly during apoptosis	22	0.214	0.792464	-0.2620884	<0.001	<0.001	0.6978587
GO:0007005	mitochondrion organization and biogenesis	137	0.09	0.9947778	0.4035484	<0.001	<0.001	0.6073059
GO:0022411	cellular component disassembly	22	0.214	0.792464	-0.2620884	<0.001	<0.001	0.6978587
GO:0022415	viral reproductive process	59	0.213	1.074234	0.2154094	<0.001	<0.001	0.4824776
GO:0030262	apoptotic nuclear changes	25	0.483	1.1064093	0.0040825	<0.001	<0.001	0.6125828
